# Medical student and tutor perceptions on active learning strategies

**DOI:** 10.1080/10872981.2019.1650565

**Published:** 2019-08-07

**Authors:** Brian Wang, Ashiq Abdul Khader

**Affiliations:** Department of Medicine, Imperial College London, London, UK

The article by Kurtz and colleagues [2] investigates the benefits of active learning for students. In the study, students were taught how to create assessments in the form of multiple-choice questions (MCQs) before questioning their perception of engagement in the activities. As medical students that have also led one-to-one and team-based teaching for medical students, we highlight in this letter the major aspects of this article and the implications this has for medical education.

A result of the study is that the students perceived that writing MCQs required more problem-solving integration compared to their preferred strategies. It has been reported in multiple studies that active learning simultaneously improves knowledge gain and recall abilities but use of both active and passive learning increases performance outcome than either method alone [1,3]. Although the MCQs did undergo editing, an interesting result would have been to identify student results from the question bank of MCQs. The creation of question banks is limited by the ability of students to critically appraise the plausible distractors. This can be affected by the number of students; a greater number of students would increase the chances of correct answers being identified but could also lead to distractors having a greater weight. This can also be affected by the time between learning of the material and the time for constructing MCQs and plausible distractors. A longer period could allow students to have a greater understanding of the material and therefore increase engagement, but reduce the benefits of the exercise.

The authors of this letter have experienced and delivered team-based learning strategies that involve construction and discussion of MCQs in a medical university environment. Both authors, however, have not had the opportunity to receive or deliver training on how to write MCQs. It would be very interesting to compare groups of students that received the training against those that have not, to see if the teaching resulted in an improvement in performance as measured by MCQ score. This would enable a quantitative understanding of whether the active learning strategy investigated in the article actually improved student performance. It would also be interesting to see if there was any difference between the two groups in the extent of editing required.

We agree that constructing MCQs in a team-based environment is a very useful tool for medical education. Much like the working life of medical professions, the discussions that occur in a multidisciplinary team largely involve determining the most appropriate managements while deprioritising alternative strategies that, like the distractors in MCQs, may not be harmful to the patient or strictly incorrect for the case but are actually not the most appropriate treatments for the patient or case.
